# Recent developments in primer design for DNA polymorphism and mRNA profiling in higher plants

**DOI:** 10.1186/1746-4811-2-4

**Published:** 2006-03-01

**Authors:** Xiaohan Yang, Brian E Scheffler, Leslie A Weston

**Affiliations:** 1 Department of Horticulture, Cornell University, Ithaca, NY 14853, USA; 2 Department of Plant Sciences, University of Tennessee, 2431 Joe Johnson Drive, Knoxville, TN 37996, USA; 3 USDA-ARS-CGRU, MSA Genomics Laboratory, 141 Experiment Station Rd., Stoneville, MS 38776, USA

## Abstract

Primer design is a critical step in the application of PCR-based technologies in gene expression and genetic diversity analysis. As more plant genomes have been sequenced in recent years, the emphasis of primer design strategy has shifted to genome-wide and high-throughput direction. This paper summarizes recent advances in primer design for profiling of DNA polymorphism and mRNA in higher plants, as well as new primer systems developed for animals that can be adapted for plants.

## Introduction

mRNA profiling is very important for identifying new genes, determining gene function and elucidating genetic networks. Differential display [1,2], cDNA amplified fragment length polymorphism (cDNA-AFLP) [3-5], and microarray [6] technologies have been employed extensively for profiling of plant mRNAs. DNA polymorphism profiling is essential for gene mapping, marker-assisted selection of crop plants, and molecular diversity studies. PCR-based techniques such as AFLP and microsatellites or simple sequence repeats (SSRs) have also played important roles in plant DNA profiling. Primers are essential components of PCR-based systems as well as modern microarray systems which utilize appropriate probes that are obtained by PCR amplification. This paper summarizes recent advances in primer design for profiling of DNA polymorphism and mRNA in higher plants, as well as new primer systems developed for animals that can be adapted for plants.

### PCR primer design in general

Understanding of primer properties is very important for primer design. The major aspects of primer properties include specificity, melting temperature (T_m_), and intra-primer or inter-primer homology. Primer specificity is mostly determined by the 3'-end sequences. It was reported that single internal mismatches had no significant effect on PCR product yield while the 3'-terminal mismatches, especially the A:A, A:G, G:A, and C:C mismatches, markedly reduced overall PCR product yield [7]. Khabar et al. [8] assessed the annealing specificity of primers in PCR reactions under different annealing temperatures (35°C, 40°C, and 45°C). They found that there were perfect matches between at least eight bases at the 3' end of the 5' primers and the target region, whereas mis-priming occurred only toward the 5' end. Therefore it is critical to include 8–10 unique bases in the 3'-end of the primer. The site-specificity of the primer can be checked by performing a sequence homology search (e.g. blastn) through all known template sequences in the public genome database such as National Center for Biotechnology Information (NCBI) [9]. To ensure specific annealing of primer to the DNA template, it is also important to avoid 4 or more G's or C's in a row in the 3'-end. T_m _is determined by primer length, GC-content and nucleotide composition. Ideally the primer will have a T_m _in the range of 50 – 65°C, random nucleotide composition, a 40–60% GC-content, and be 18 – 30 bases long. The intra-primer or inter-primer homology should be kept as low as possible to avoid formation of hairpin structures (>3 bp complementarity within primer) or primer dimers (>3 bp complementarity between primers) which will interfere with annealing of primer to the DNA template [10]. Up to date a lot of programs have been developed for primer design. Here we introduce some web-based and stand-alone programs which are free for public use (Table 1).

**Table 1:** Free programs for PCR primer design.

***Name***	***Web source***	***Ref.***	Note*
	**General primer design**		
Primer3		[63]	W
GeneFisher		[64]	W
Primo Pro 3.4		[65]	W
PRIMO		[66]	W
FastPCR		[67]	S
	**Primer design based upon multi-alignments**		
Primaclade		[68]	W
CODEHOP		[69]	W
PriFi		[70]	W
	**Primer design for single-nucleotide polymorphism (SNPs)**		
IRA-PCR		[71]	W
SNP_Primers		[72]	W
Primo SNP 3.4		[65]	W
	**Primer design for Simple Sequence Repeat (SSR)**		
SSR Finder		[73]	W
	**Primer design for Microarrays**		
ProbeWiz		[74]	W
ROSO		[75]	W
OligoWiz 2		[76]	S
Picky		[77]	S
	**Oligonucleotide properties calculation**		
Oligo Analyzer		[78]	W
Poland server		[79]	W
NetPrimer		[80]	W
dnaMATE		[81]	S/W

*Note: W = Web-based; S = Standalone.

### Primer design for mRNA profiling

#### Primers for differential display

In traditional differential display (DD), cDNAs are amplified with 3' one-base anchored oligo-dT primers and short 5' arbitrary primers designed to be maximally different in their 7-base 3' sequence while the six 5' bases are fixed (Table 2). Targeted 5' primers can be designed to match a given mRNA at a position that allows detection of the reverse transcriptase PCR (RT-PCR) product on a DD gel. Combining a targeted 5' primer with the three 3' one-base anchored oligo-dT primers (in different reactions) should result in display of a fragment of the expected size in one of the combinations [11]. However, Jorgensen et al. [11] reported that successful display of a targeted mRNA was only achieved in 50 – 60% of the trials, suggesting that display of a band was mainly dependent on the ability of that cDNA to compete in the competitive PCR reaction that is the basis for DD. Recently, it was shown that DD failed to display the *gadd45 *mRNA in hamster despite the use of two *gadd45*-specific primers and the high level of *gadd45 *transcript in the RNA sample [12]. Thus it seems difficult to predict whether or not a given primer will detect a specific transcript, even if abundant, in an uncharacterized cDNA population [6].

**Table 2:** Primer design for differential display.

***Traditional differential display system***[16]
**Forward**
5'-AAGCTTXXXXXXX-3' The XXXXXXX was designed to target the mRNA sequences with a good coverage of mRNA species in the sample.
**Reverse**
5'-AAGCTTTTTTTTTTTTTA-3'
5'-AAGCTTTTTTTTTTTTTA-3'
5'-AAGCTTTTTTTTTTTTTG-3'
***Annealing control primer system***[14]
**Forward**
5'-GTCTACCAGGCATTCGCTTCATIIIIIXXXXXXXXXX-3' The XXXXXXXXXX was designed to target the mRNA sequences with a good coverage of mRNA species in the sample.
**Reverse**
5'-CTGTGAATGCTGCGACTACGATIIIIITTTTTTTTTTTTTTT-3'
***Multiplex differential display system ***[15]
**Forward**
5'-CTTNNXXXXXXXX-3' (N = A, C, G, or T) The XXXXXXXX was designed to target the mRNA sequences with a good coverage of mRNA species in the sample.
**Reverse**
5'-AAGCTTTTTTTTTTTTTC-3'
5'-AAGCTTTTTTTTTTTTTG-3'
5'-AAGCTTTTTTTTTTTTTA-3'

Hwang et al. [13] developed an annealing control primer (ACP) system that is comprised of a tripartite structure with a polydeoxyinosine [poly(dI)] linker between the 3' end target core sequence and the 5' end non-target universal sequence. This ACP linker prevents annealing of the 5' end non-target sequence to the template and facilitates primer hybridization at the 3' end to the target sequence at specific temperatures, resulting in a dramatic improvement of annealing specificity. This system was recently adapted for the identification of differentially expressed genes involved in mouse development [14]. The primer design of ACP is shown in Table 2. This system could be easily adapted for mRNA profiling in plants by substituting the 3' end animal-targeting sequences for those targeting plant mRNAs. To evaluate our ability to improve annealing specificity, our laboratory also designed a multiplex DD system, in which the 5' primers were designed as 5'-CTTNN-eight mRNA specific bases – 3' (N = A, C, G, or T) (Table 2). The rationale for this primer structure is that in a PCR reaction one 3' primer is used with a mixture of sixteen 5' primers that share the eight 3' bases but differ in the "N" wobble sites. Under high stringency PCR conditions, each of the sixteen 5' primers binds preferentially to a group of mRNA species that perfectly match the eight 3' bases of the 5' primers, reducing competition for amplification among cDNAs, and consequently increasing the chance of detecting a specific transcript by a given 5' primer [15]. Both the primers in ACP and the primers designed by ourselves are longer than those in the original DD [16]. Generally, the annealing temperature increases with the length of the primer. With the original DD annealing temperature, 40–50°C [17], the size changes of these modified primers may result in the improvement of PCR efficiency so that DD could produce more strong bands in the DD gels.

It was estimated that DD can detect approximately 96% of expressed genes in a cell utilizing the three different oligo-dT primers in combination with 80 arbitrary primers [16]. This estimation was based on the hypothesis that genes have random nucleotide distributions. However, experimental approaches, as well as computer analysis of genomic sequences, have revealed that there is large variation in base composition between regions in the same genome or between different genomes [18]. The nucleotide distribution within genes is not random [19]. Therefore, random design is not the best approach for creating DD primers. It would be more logical to use a bioinformatical approach for custom design of DD primers based on mRNA sequence information. We designed a set of eight-base sequences targeting plant mRNAs [15]. Specifically, an initial pool of 1,292 eight-base sequences was established based on the analysis of codon usage in eight plant species that included four dicots (*Arabidopsis thaliana*, *Lycopersicon esculentum*, *Medicago sativa*, *Nicotiana tabacum*) and four monocots (*Oryza sativa*, *Sorghum bic*olor, *Triticum aestivum*, *Zea mays*). The initial pool of 5' primers was screened against the database At (11,583 *A. thaliana *mRNA sequences) for perfect matches between primers and mRNA sequences in the region of 400 – 1,500 nt from the 3' end. The mRNAs were divided into two groups, with each mRNA having one, and more than one primer matches, respectively. The sequences matching the mRNAs that had one primer match each were selected into the primer set for mRNA profiling, which contained 142 primers (Table 3). The selected primer set was tested on databases At800 (9,370 *A. thaliana *mRNA sequences derived from database At by removing sequences with a length of ≤800 nt), Odi800 (650 mRNA sequences of >800 nt from the three dicots: *L. esculentum*, *M. sativa*, *N. tabacum*), and Mon800 (1,081 mRNA sequences of >800 nt from the four monocots: *O. sativa*, *S. bic*olor, *T. aestivum*, *Z. mays*) by a search for perfect matches between primers and mRNA sequences in the region of 400 – 1,500 nt from the 3' end. The primer set matched ~91% of mRNAs in each of the three databases. Since these databases contain mRNA sequences of diverse plant species including both monocots and dicots, it is also likely that this primer set would generate good coverage of mRNAs in a variety of other plant species as well. There was no major difference in the number of primer matches per mRNA among the three databases tested, with an average of about three primer matches per mRNA. This sampling redundancy is close to an estimation given by calculations using equation P(0) = e^-μ^, where μ represents the sampling redundancy and P(0) represents the probability of missing an mRNA by primers [20]. According to this equation, each mRNA species needs to be matched (or sampled) 2.4 [= -LN(1-0.91)] times on average by the oligos to achieve at least 91% coverage of an mRNA database. Also, there was no significant difference observed in the mRNA-matching frequency per primer among the three databases evaluated. On average each primer matched ~2% of mRNAs in each of the databases.

**Table 3:** The 3' eight mRNA specific bases for the 5' primer set for Multiplex DD [15].

***ID***	***Sequence***	***ID***	***Sequence***	***ID***	***Sequence***	***ID***	***Sequence***
**1**	TACTCCCT	**37**	CAAAGGAC	**73**	TCTTGGGT	**109**	CCAAGAGT
**2**	ATCTCCGA	**38**	GATAGGAG	**74**	ACATGGAC	**110**	GCTAGAAC
**3**	TCTTCCGA	**39**	CAAAGGAG	**75**	CAATGGAG	**111**	ACCAGAAC
**4**	TCATCCGA	**40**	TCTAGGAG	**76**	CTTTGGTC	**112**	AGCAGAAC
**5**	AGATCCGA	**41**	AAGAGGTC	**77**	AGTTGGTC	**113**	GAGAGAAG
**6**	GATTCCGT	**42**	CAAAGGTC	**78**	AAGTGGTG	**114**	CAGAGAAG
**7**	ATGTCCGT	**43**	AGAAGGTC	**79**	GAATGGTG	**115**	GGAAGAAG
**8**	AACTCCGT	**44**	AAGAGGTG	**80**	AACTGGTG	**116**	GTGAGATC
**9**	GAATCCAC	**45**	ACAAGGTG	**81**	AGTTGGTG	**117**	CTCAGATC
**10**	TTCTCCAC	**46**	AAGAGCCA	**82**	AAGCACCA	**118**	ACCAGATC
**11**	ATCTCCAC	**47**	CAAAGCCA	**83**	TTCCACCA	**119**	GCAAGATG
**12**	GTTTCCAG	**48**	ATCAGCCA	**84**	CATCACCA	**120**	GTCCATCA
**13**	ATCTCCAG	**49**	AGTAGCCA	**85**	AACCACCT	**121**	GAGCATCT
**14**	AAGTCCTC	**50**	TTCAGCCT	**86**	CTTCACCT	**122**	GCTCATCT
**15**	GATTCCTC	**51**	CTTAGCCT	**87**	TCTCACCT	**123**	GCTCATGT
**16**	ATGTCCTC	**52**	ACAAGCCT	**88**	CAACACGA	**124**	AGCCATGT
**17**	GTTTCCTC	**53**	AAGAGCGA	**89**	CTTCACGA	**125**	GACCATAC
**18**	AACTCCTC	**54**	TACAGCGA	**90**	ACACACGA	**126**	CTCCATAC
**19**	AGATCCTC	**55**	TTCAGCAC	**91**	CTTCACGT	**127**	CACCATTC
**20**	TTCTCCTG	**56**	ATCAGCAG	**92**	GAACACAC	**128**	GCTCATTG
**21**	AACTCCTG	**57**	TACAGCAG	**93**	GTTCACAG	**129**	CTCCATTG
**22**	ATCAGGCA	**58**	AGAAGCAG	**94**	CTTCACTC	**130**	GAGAGTCA
**23**	AAGAGGCT	**59**	AAGAGCTC	**95**	CTTCACTG	**131**	CCAAGTCT
**24**	ATGAGGCT	**60**	ATGAGCTC	**96**	CCTACACT	**132**	GTCAGTCT
**25**	CAAAGGCT	**61**	ATCAGCTC	**97**	CCAACAGA	**133**	GAGAGTGT
**26**	TTGAGGCT	**62**	TTGAGCTC	**98**	TGGACAGT	**134**	GTGAGTGT
**27**	TCTAGGCT	**63**	ACAAGCTC	**99**	CTCACAAC	**135**	CTCAGTAC
**28**	TCAAGGCT	**64**	TCAAGCTG	**100**	GTCACAAC	**136**	GGTAGTTC
**29**	AGAAGGCT	**65**	CATAGCTG	**101**	GGAACATC	**137**	CTGAGTTC
**30**	ATGAGGGA	**66**	CTTTGGCA	**102**	CACACATC	**138**	GTCAGTTC
**31**	TCAAGGGA	**67**	AGATGGCA	**103**	GTGACATG	**139**	GCTAGTTG
**32**	AAGAGGGT	**68**	GAATGGCT	**104**	CCAAGACA	**140**	CTCAGTTG
**33**	ATGAGGGT	**69**	TCATGGCT	**105**	ACCAGACT	**141**	TCCAGTTG
**34**	TTCAGGGT	**70**	AGATGGCT	**106**	GGAAGAGA	**142**	AGCAGTTG
**35**	TCTAGGGT	**71**	AGTTGGCT	**107**	GGTAGAGA		
**36**	GATAGGAC	**72**	CAATGGGT	**108**	CAGAGAGT		

### Primers for cDNA-AFLP

In cDNA-AFLP, cDNA samples are digested with two different restriction enzymes, adapters are attached to the specific ends of the resulting fragments, and the fragments are amplified using primers homologous to the adaptors with an extension of additional selective nucleotides. Thus, for each selective primer pair only the fragments whose ends match the primer extensions get amplified and these fragments form a pool. Finally, the fragments in each pool are separated by electrophoresis [21]. The primer design for cDNA-AFLP depends on the choice of restriction enzymes and 3'selective sequences. Unfortunately, one pair of enzymes does not in practice produce a fragment for every cDNA molecule that could be amplified and detected by electrophoresis. The fragments generated from a particular cDNA can be too long or too short to be revealed by electrophoresis. One pair of restriction enzymes generally covers up to two-thirds of the transcripts in a species [4,21,22]. In cDNA-AFLP performed with plant samples, two selective bases on the 3' end of each primer are required to give a scorable banding pattern, giving a total of 256 (16 × 16) possible primer combinations [22].

Recently, Wang and Bughrara [5] found that for *Festuca *species, restriction enzyme *Nsp*I coupled with *Taq*I generated a much higher number of transcript-derived fragments than the commonly used enzyme pair of *Eco*RI and *Taq*I. The enzyme *Nsp*I has two degenerate bases in its recognition sequence (RCATGY). This enzyme can cut the cDNA more frequently than *Eco*RI, and generate more bands in the cDNA-AFLP gels. An additional advantage of using enzyme pair of *Nsp*I and *Taq*I is that the possible selective primer combinations were 128 (8 × 16), only half of those for *Eco*RI/*Taq*I (16 × 16). To achieve a 90% coverage of mRNAs in a species, it is necessary to increase the number of enzyme combinations up to 4 [21]. In a cDNA-AFLP system with 4 enzyme combinations and two selective bases on the 3' end of each primer, there are 1,024 (4 × 256) possible primer combinations. PCR analysis of all the 1,024 possible primer combinations would be time-consuming and costly. In addition, a portion of the mRNA species could be sampled by two or more enzyme combinations, resulting in a somewhat wasteful usage of resources. Fortunately, several computer programs have recently been developed to perform *in silico *simulation of cDNA-AFLP using available sequencing data [21,23,24]. With the assumption that the real target genome has roughly the same characteristics as the sequence data available [21], it is possible to find appropriate enzyme combinations to increase mRNA coverage while decreasing selective primer combinations by reducing redundant coverage of the same mRNA species. This could be achieved by simulating cDNA-AFLP *in silico *for the available genome sequencing data in *A. thaliana *[25], *O. sativa *[26-28] and *Populus trichocarpa *[29] as well as plant EST data in the public domains.

### Primers for microarray probes

DNA microarrays provide powerful tools for global mRNA profiling. Despite widespread use, recent studies have demonstrated discordance among data produced by different microarray platforms and approaches [30-33]. For example, Tan et al. [32] reported that from a set of 185 common genes in PANC-1 cells, only four behaved consistently on three major commercial microarray platforms from Affymetrix, Agilent and Amersham. One major reason for this is due to the fact that probes have not generally been designed in the past for specificity with gene-splice variants. It is encouraging that companies are now beginning to make arrays specific to different splice variants [31,34,35]. The discordance among different microarray platforms can also be caused by cross-hybridization of highly similar sequences [34]. Possible choices of probe types include spotted cDNA sequences or PCR products, several hundred to thousand base pairs in length, short (25–30 mer) oligonucleotides or longer (60–70 mer) oligonucleotide reporters [36]. Based on theoretical considerations that were confirmed experimentally, it appears that 150-mer is the optimal probe length for expression measurement [37], and thus PCR primers can be designed to amplify the 150-mer gene-specific probes.

Cross-species comparisons of gene expression are important for identifying functionally related genes, because if a set of genes displays similar expression patterns in several species, the probability that the genes are functionally related, rather than co-expressed by chance, increases. Microarray experiments using probes covering a whole transcriptome are expensive to initiate, and a major part of the cost arises from the synthesis of gene-specific PCR primers or hybridization probes [38]. Andersson et al. [38] developed a method to reduce the number of primers required to amplify the genes of two different genomes. In this method, regions of high sequence similarity were identified, and from these regions PCR primers shared between the genomes were selected, such that either one or, preferentially, both primers in a given PCR were used for amplification from both genomes. This method could be used to design PCR primers for amplification of microarray probes shared by *A. thaliana *and *P. trichocarpa*, or *A. thaliana *and *O. sativa*, or *P. trichocarpa *and *O. sativa*.

### Primer design for DNA profiling

#### Primers for sequence-related amplified polymorphism (SRAP)

Recently, a series of SRAP primers were designed by Li and Quiros [39] for the amplification of open reading frames (ORFs). This system is based on two-primer amplification. The 17 or 18-mer primers consist of the following elements: core sequences, which are 13 – 14 bases long, where the first 10 or 11 bases starting at the 5' end are sequences of no specific constitution ("filler" sequences) followed by the sequence CCGG in the forward primer and AATT in the reverse primer. The core is followed by three selective nucleotides at the 3' end. The filler sequences of the forward and reverse primers must be different from each other and can be 10 or 11 bases long. The SRAP system has been successfully utilized to profile DNA polymorphism in turf grass species [40], tomato (*Lycopersicon esculentum *L. Mill.) [41], and squash (*Cucurbita moschata*) [42].

#### Primers for sequence-specific amplification polymorphism (SSAP)

SSAP is a multiplex amplified fragment length polymorphism (AFLP)-like technique that displays individual retrotransposon insertions as bands on a sequencing gel. Retrotransposons are mobile genetic elements that accomplish transposition via an RNA intermediate that is reverse transcribed before integration into a new location within the host genome. They are ubiquitous in eukaryotic organisms and constitute a major portion of the nuclear genome (often more than half of the total DNA) in plants [43]. It has been successfully used for profiling of DNA polymorphism in many crops such as barley (*Hordeum vulgare*) [44], *M. sativa *[45], and sweetpotato (*Ipomoea batatas *(L.) Lam.) [46]. To adapt conventional SSAP method to high-throughput situations, Tang et al. [47] developed and optimized a fluorescent multiplex PCR system for simultaneous selective amplification of Ty1-copia retrotransposon-based SSAPs, followed by capillary electrophoresis.

#### Primers for simple sequence repeat (SSR)

Recently, Robinson et al. [48] developed a computer program to identify and design PCR primers for amplification of SSR loci based on available DNA sequence information. SSR primers have been designed using publicly available expressed sequence tags (ESTs) in barley [49,50], almond (*Prunus communis *Fritsch.) [51], peach (*P. persica *(L.) Batsch.) [51], *T. aestivum *[52], and *O. sativa *[52]. These SSRs are useful as molecular markers because their development is inexpensive, they represent transcribed genes and their putative function can often be deduced by a homology search [53]. SSRs have been the backbone to creating molecular maps for a number of years.

Chung and Staub [54] developed a set of consensus chloroplast primer pairs for simple sequence repeats (ccSSRs) from *N. tabacum *chloroplast sequences. All primer pairs produced amplicons after PCR employing chloroplast DNA from members of the *Cucurbitaceae *(six species) and *Solanaceae *(four species). Sixteen, 22 and 19 of the initial 23 primer pairs were successively amplified by PCR using template DNA from species of the *Apiaceae *(two species), *Brassicaceae *(one species) and *Fabaceae *(two species), respectively. Twenty of the 23 primer pairs were also functional in three monocot species of the *Liliaceae *(onion and garlic), and the *Poaceae *(oat). ccSSR primers were strategically "recombined" and were referred to correctly as recombined consensus chloroplast primers (RCCP) for PCR analysis of cucumber DNA.

### Target-specific PCR primers

Gawel et al. [55] developed a semi-specific PCR system in which primers were designed to target the semi-conservative sequences of the intron-exon junction. The most informative primers were selected from among the exon targeting (ET) and intron targeting (IT) primers, 12 to 18 bases in length. Also, Holland et al. [56] developed PCR primer pairs that target exons, introns, promoter regions in *Z. mays *and introns as well as repeat sequences in *Avena sativa*. Most recently, Hu and Vick [57] developed a primer system called target region amplification polymorphism (TRAP). This system uses 2 primers of 18 nucleotides each to generate markers. One of the primers, the fixed primer, is designed from the targeted EST sequence in the database; the second primer, the arbitrary primer, is an arbitrary sequence with either an AT- or GC-rich core to anneal with an intron or exon, respectively. The TRAP technique, taking advantage of the availability of sequence information, should be useful in plant genomics research involved in marker-trait association [57].

### Primers for single-nucleotide polymorphism (SNPs)

**Figure 1:**
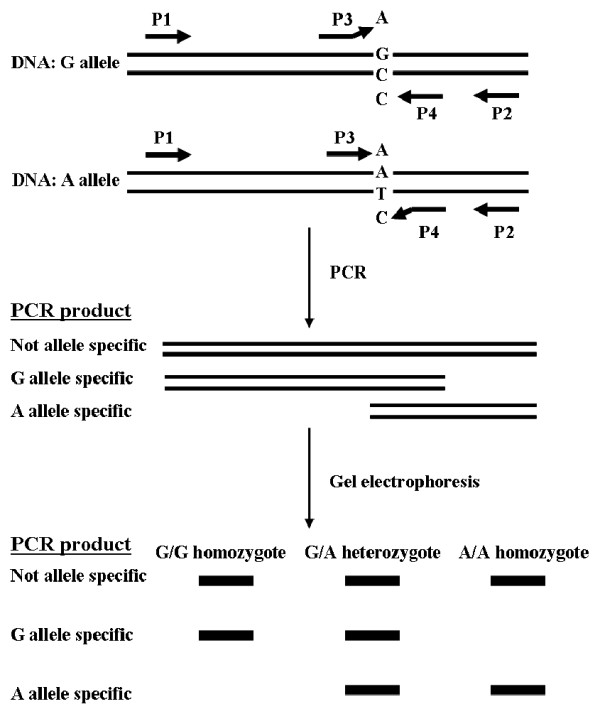
**Diagrammatic presentation of the tetra-primer ARMS-PCR method for SNP identification (Redrawn from [58])**. Two allele-specific amplicons are generated using two pairs of primers, one pair (P1 and P4) producing an amplicon representing the G allele and the other pair (P2 and P3) producing an amplicon representing the A allele. By positioning the two outer primers (P1 and P2) at different distances from the polymorphic nucleotide, the two allele-specific amplicons differ in length, allowing them to be discriminated by gel electrophoresis.

Ye et al. [58] established an efficient procedure for genotyping single nucleotide polymorphisms, named tetra-primer ARMS-PCR, which employs two primer pairs to amplify, respectively, the two different alleles of a SNP in a single PCR reaction. ARMS-PCR has been used for barley SNP genotyping [59]. The ARMS-PCR primer system is illustrated in Figure 1. Also, Kota et al. [50] developed SNP primer pairs for barley based on available EST database. Recently, a computer program was developed to automate the primer design process for SNP analysis [60]. Recently, Rudd et al. [61] created a database resource, PlantMarkers, to predict, analyze and display various molecular markers including SNP and SSR for over 50 plant species. This database will greatly facilitate primer design for profiling of DNA polymorphisms using SNP and SSR in the future.

### Universal rice primer (URP)

Repeat-based PCR strategies such as microsatellites are also potentially very useful for DNA polymorphism profiling. Recently, Kang et al. [62] developed a primer system, referred to as the universal rice primer (URP), based on a repetitive DNA fragment (pKRD) in rice. Forty 20-mer primers were randomly designed from the entire pKRD fragment, with the idea that short oligomers complementary to primers are well dispersed within the rice genome. Twelve primers listed in Table 4 produced characteristic fingerprints from diverse genomes of 14 plant species: *O. sativa*, *Z. mays*, barley, bamboo (*Phyllostachys spp*.), oat (*A. sativa*), soybean (*Glycine max *L.), chinese cabbage (*Brassica rapa *var. *pekinensis*), pumpkin (*Cucurbita pepo *L.), cucumber (*Cucumis sativa *L.), spinach (*Spinaceae oleracea *L.), pepper (*Capsicum annuum *L.), garlic (*Allium sativum *L.), *N. tabacum*, *A. thaliana*, 7 animal species and 6 microbial species, indicating its universal applicability.

**Table 4:** Oligonucleotide sequences of 12 URP primers (Adapted from [62]).

***Primers***	***Sequences (5'-3')***
URP1F	ATCCAAGGTCCGAGACAACC
URP2F	GTGTGCGATCAGTTGCTGGG
URP2R	CCCAGCAACTGATCGCACAC
URP4R	AGGACTCGATAACAGGCTCC
URP6R	GGCAAGCTGGTGGGAGGTAC
URP9F	ATGTGTGCGATCAGTTGCTG
URP13R	TACATCGCAAGTGACACAGG
URP17R	AATGTGGGCAAGCTGGTGGT
URP25F	GATGTGTTCTTGGAGCCTGT
URP30F	GGACAAGAAGAGGATGTGGA
URP32F	TACACGTCTCGATCTACAGG
URP38F	AAGAGGCATTCTACCACCAC

## Conclusion

In the post-genomics era, recent availability of DNA sequence data has fostered the further development of DNA/mRNA profiling technologies that exhibit enhanced genome-wide coverage and improved targeting accuracy. Recent trends related to primer design include the following:

1) Optimization of primer structure or probe properties for increased specificity of primer (or probe) hybridization to the target sequence in PCR reactions (or microarray analysis).

2) Increased genome-wide coverage with minimum primer numbers and reduced sampling redundancy.

3) Novel primer design for amplification of microarray probes specific to gene-splice variants for more accurate mRNA profiling.

Recent innovations have led to more cost-effective and successful profiling studies and greater ease in subsequent purification of gene fragments. This review is intended not only to help scientists to update their knowledge of primer design for DNA polymorphism and mRNA profiling in higher plants, but also to increase their interests in making technical improvements using plant genomics and bioinformatics approach.

## Competing interests

The author(s) declare that they have no competing interests.

## Authors' contributions

XY carried out the design of the modified primers for differential display, and drafted the manuscript. BS and LW conceived of the study, and participated in its design and coordination. All authors read and approved the final manuscript.
